# Airway obstruction, dynamic hyperinflation, and breathing pattern during incremental exercise in COPD patients

**DOI:** 10.1002/phy2.222

**Published:** 2014-02-07

**Authors:** Bente Frisk, Birgitte Espehaug, Jon A. Hardie, Liv I. Strand, Rolf Moe‐Nilssen, Tomas M. L. Eagan, Per S. Bakke, Einar Thorsen

**Affiliations:** 1 Centre for Evidence‐Based Practice, Bergen University College, Bergen, Norway; 2 Department of Clinical Science, University of Bergen, Bergen, Norway; 3 Department of Global Public Health and Primary Care, University of Bergen, Bergen, Norway; 4 Department of Physiotherapy, Haukeland University Hospital, Bergen, Norway; 5 Department of Thoracic Medicine, Haukeland University Hospital, Bergen, Norway; 6 Department of Occupational Medicine, Haukeland University Hospital, Bergen, Norway

**Keywords:** Chronic obstructive pulmonary disease, exercise, inspiratory capacity, spirometry

## Abstract

Ventilatory capacity is reduced in chronic obstructive pulmonary disease (COPD) patients. Tidal volume (*V*_T_) is lower and breathing frequency higher at a given ventilation (*V*_E_) compared to healthy subjects. We examined whether airflow limitation and dynamic hyperinflation in COPD patients were related to breathing pattern. An incremental treadmill exercise test was performed in 63 COPD patients (35 men), aged 65 years (48–79 years) with a mean forced expiratory volume in 1 sec (FEV_1_) of 48% of predicted (SD = 15%). Data were averaged over 20‐sec intervals. The relationship between *V*_E_ and *V*_T_ was described by the quadratic equation *V*_T_ = *a* + *bV*_E_ + *cV*_E_^2^ for each subject. The relationships between the curve parameters b and c, and spirometric variables and dynamic hyperinflation measured as the difference in inspiratory capacity from start to end of exercise, were analyzed by multivariate linear regression. The relationship between *V*_E_ and *V*_T_ could be described by a quadratic model in 59 patients with median *R*^2^ of 0.90 (0.40–0.98). The linear coefficient (b) was negatively (*P* = 0.001) and the quadratic coefficient (c) positively (*P* < 0.001) related to FEV_1_. Forced vital capacity, gender, height, weight, age, inspiratory reserve volume, and dynamic hyperinflation were not associated with the curve parameters after adjusting for FEV_1_. We concluded that a quadratic model could satisfactorily describe the relationship between *V*_E_ and *V*_T_ in most COPD patients. The curve parameters were related to FEV_1_. With a lower FEV_1_, maximal *V*_T_ was lower and achieved at a lower *V*_E_. Dynamic hyperinflation was not related to breathing pattern when adjusting for FEV_1_.

## Abstract (precis)

The relationship between minute ventilation and tidal volume during incremental treadmill exercise could be described by a quadratic model in COPD patients. The curve parameters were related to airway obstruction, but not to dynamic hyperinflation or inspiratory reserve volume by the end of exercise.

## Introduction

Exertional dyspnea is one of the main factors limiting physical activity in patients with chronic obstructive pulmonary disease (COPD) (O'Donnell and Webb 1993; Maltais et al. 2005; Nici et al. 2006). At a given expired minute ventilation (*V*_E_), the tidal volume (*V*_T_) is lower and the breathing frequency (*B*_f_) higher in patients having COPD compared to healthy subjects (Palange et al. 2007). The maximal ventilatory capacity is reduced (Gallagher 1994), and is closely related to forced expired volume in 1 sec (FEV_1_) (Clark et al. 1969; Potter et al. 1971).

The mechanism for the ventilatory limitation in COPD is related to expiratory flow limitation and lung hyperinflation. The time constant for the lung, which is the product of resistance and compliance, is increased and during progressively higher ventilatory demands, expiration may not be completed before the drive for the next inspiration starts (Hyatt 1983). End‐expiratory lung volume increases, and breathing takes place at a higher lung volume where both resistance and compliance are lower. The effect of these changes in lung volume, resistance, and compliance is a shorter time constant allowing complete respiratory cycles, but it is at the cost of a higher work of breathing (Hyatt 1983; O'Donnell and Webb 1993). The *V*_T_ is constrained by total lung capacity (TLC) on the inspiratory side. The inspiratory reserve volume (IRV) falls by increasing *V*_T_. Expiratory constraints are more complex, influenced by the increased time constant and inspiratory drive (Peters et al. 2006).

The relationship between *V*_E_ and *V*_T_ during incremental exercise can be described by three phases (Gallagher et al. 1987). In the first phase, there is an almost linear relationship between *V*_E_ and *V*_T_. In the second phase, the increase in *V*_E_ is mainly caused by an increase in *B*_f_ and a smaller increase in *V*_T_. In the third phase, the increase in *V*_E_ is caused by an increase in *B*_f_ only, and by the end of this phase there can be a fall in *V*_T_ (Gallagher et al. 1987). The relationship between *V*_E_ and *V*_T_ has previously been described by various methods such as the maximal *V*_T_ (*V*_Tmax_) or the plateau of *V*_T_ and the inflection point (O'Donnell et al. 2006), *V*_Tmax_ and *V*_T_ at a *V*_E_ of 30 L/min (Cotes 1972), *V*_T_ at given fractions of peak *V*_E_ (Neder et al. 2003), and the slope and intercept of the first part of the response (Hey et al. 1966). However, neither of these methods account for the curvilinearity of the response. In young healthy subjects, the individual relationship between *V*_E_ and *V*_T_ has been described satisfactorily by a quadratic (Kalsas and Thorsen 2009) and a logarithmic (Naranjo et al. 2005) relationship, but it is not known whether these models are applicable for the general population or patients with lung disease.

The aim of this cross‐sectional study was to examine whether a quadratic model could satisfactorily describe the relationship between *V*_E_ and *V*_T_ during exercise in COPD patients. The hypothesis was that the curve parameters of the quadratic model, which describe the breathing pattern, were related to FEV_1_, IRV, and dynamic hyperinflation.

## Methods

### Subjects

Of the 433 patients included in the Bergen COPD Cohort study (Eagan et al. 2010), 89 patients participated in a pulmonary rehabilitation program during the first 2 years of follow‐up in 2006–2008. In 2011–2012, 63 of these patients were available for a cardiopulmonary exercise test on a treadmill. The remaining 26 patients were deceased or disabled.

The included patients had clinically stable COPD in Global Initiative for Chronic Obstructive Lung Disease (GOLD) (Rabe et al. 2007) stages II–IV and age between 48 and 79 years. Thirty‐two subjects were in stage II, 23 in stage III, and eight in stage IV. All patients had a smoking history of ≥10 pack‐years, a postbronchodilation FEV_1_ to forced vital capacity (FVC) ratio <0.7 and a postbronchodilator FEV_1_ <80% of predicted value according to Norwegian reference values (Johannessen et al. 2006). Patients with inflammatory disorders like rheumatoid arthritis, systemic lupus erythematosus or other connective tissue disorders, inflammatory bowel disease, and any active cancer in the last 5 years were not included in the Bergen COPD Cohort study. Exclusion criteria for exercise testing were major cardiovascular disorders, a partial pressure of oxygen in arterial blood less than 8 kPa at rest, or exacerbations that required medical treatment during the last 4 weeks prior to testing. The patients were examined by a physician prior to exercise testing.

### Ethics

The Western Norway Regional Research Ethics Committee approved the study. Participation in the study was voluntary. Written and oral information was given and written consent was obtained prior to inclusion.

### Spirometry

Spirometry was conducted on a Viasys Masterscope (Viasys, Hoechberg, Germany) before the exercise test according to the ATS/ERS Standardization of Lung Function Testing (Miller et al. 2005). The FVC and FEV_1_ were taken as the highest values from at least three satisfactory expiratory maneuvers. The spirometer was calibrated before each test with a 3‐L calibration syringe. The body mass index (BMI) was calculated as the body mass divided by the square of height.

### Cardiopulmonary exercise test

The patients completed an incremental exercise test to their symptom‐limited maximum on a treadmill (Woodway, model: PPS 55 med Weiss, Weil am Rhein, Germany). The exercise protocol was a modified Bruce protocol (Bruce 1971; Bruce et al. 1973), and started with rest in the standing position for 2 min. The warm‐up phase lasted for 1 min with a walking speed of 1.5 km/h. Blood pressure, electrocardiography (GE Healthcare, Cardio Soft EKG, Freiburg, Germany) and pulse oximetry were monitored at rest, continuously during the test and for 3 min into the recovery phase. A tight‐fitting oronasal mask was adjusted to each patient and checked for leaks before starting the exercise. The integrated exercise testing system (Care Fusion, V_max_ Spectra 229, Hochberg, Germany), was calibrated every morning and immediately before each test. The *V*_T_, *B*_f_, oxygen uptake (VO_2_), carbon dioxide production (VCO_2_), and heart rate (HR) were measured on a breath by breath basis and averaged over 20‐sec intervals. *V*_E_ and *V*_T_ were corrected to the body temperature pressure saturated (BTPS) condition, and VO_2_ and VCO_2_ to the standard temperature pressure (STPD) condition.

The patients graded their level of dyspnea and leg discomfort by the Borg CR10 Scale (Borg 1998) before the test started, every second minute during the test, and at peak exercise. In order to measure hyperinflation during exercise, serial measurements of inspiratory capacity (IC) as described by O'Donnell and Webb (1993) were performed. Measurements were taken before the start of exercise, every second minute during exercise and at peak exercise. Patients who had a decrease in IC from rest to peak exercise (ΔIC) ≥0.4 L (O'Donnell and Laveneziana 2006a) were characterized as hyperinflators, the rest as nonhyperinflators. We also calculated ΔIC adjusted for resting IC (ΔIC_adj._). A reduction in ΔIC_adj._ ≥20% was used as cut‐off limit for comparison of the subjects (O'Donnell and Laveneziana 2006b). The IRV was calculated as the difference between IC at the end of the test minus the preceding *V*_T_.

### Statistical analyses

Descriptive statistics were used to characterize the study population (mean, standard deviation [SD], and percent). Independent samples t‐tests were used to compare continuous variables and Pearson χ^2^ tests for categorical variables. The relationship between *V*_E_ and *V*_T_ was described for each individual by the quadratic model *V*_T_ = *a* + *bV*_E_ + *cV*_E_^2^. The goodness of fit for the individual patient‐specific regression analysis was evaluated by the adjusted coefficient of determination (adjusted *R*^2^) and the *F*‐statistic. For the latter a *P*‐value <0.05 was required for inclusion of the patient in further analysis. The relationship between the estimated curve parameters in the quadratic model, the intercept (*a*), the slope (*b*), and the curvature (*c*), respectively, and age, gender, height, weight, FEV_1_, FVC, IRV, and ΔIC_adj._ were analyzed by bivariate and multivariate linear regression analysis. IC at rest was also used in the multivariate analysis, but was not significant and therefore excluded from the final model.

The goodness of fit of the quadratic model was compared with the goodness of fit by a hyperbolic (inverse) model of the form *V*_T_ = *a* + *bV*_E_^∢1^.

Estimated regression coefficients are presented with 95% confidence intervals (CI) and *P*‐values. The significance level was set at 0.05. The data analyses were performed using IBM SPSS Statistics 21 (SPSS Inc. Chicago, IL).

## Results

**Figure 1.**
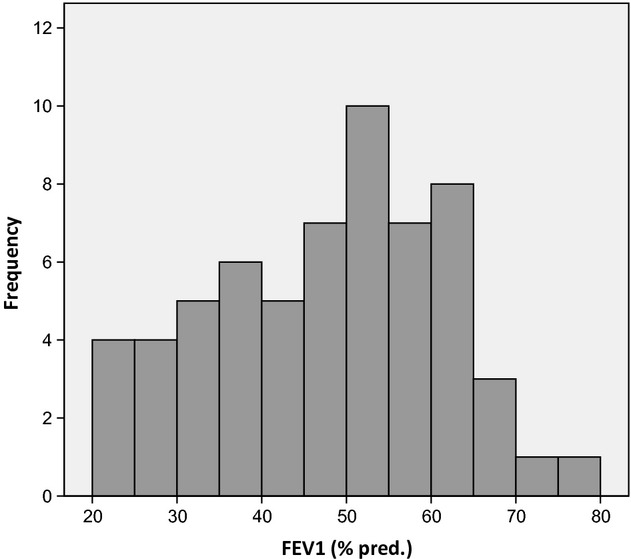
The distribution of FEV_1_ in% of predicted.

**Figure 2.**
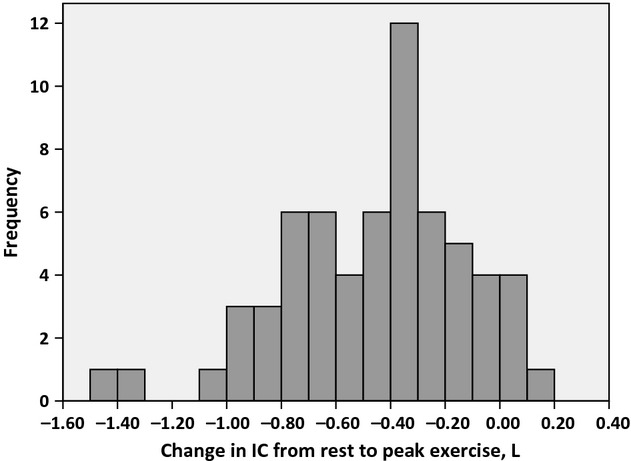
The distribution of change in inspiratory capacity (IC) from rest to peak exercise.

Subject characteristics and resting pulmonary function measurements are summarized in Table 1. The patients were airflow limited with a mean FEV_1_ of 48% of the predicted value (Fig. 1). Thirty‐two patients were categorized as hyperinflators with a ΔIC ≥0.4 L and 31 as nonhyperinflators. The same result was demonstrated when using a ΔIC_adj._ ≥20% as cut off. The distribution of ΔIC from rest to peak exercise is illustrated in Figure 2. Of the hyperinflators 72% were men, and of the nonhyperinflators 39%. The peak responses to treadmill exercise are presented in Table 2. There were no significant differences in exercise time, VO_2peak_, VCO_2peak_, V_Epeak_, HR_peak_, Borg scores, and desaturation between the hyperinflators and nonhyperinflators. Fifty‐three (84%) of the patients stopped exercise due to dyspnea or dyspnea in combination with leg discomfort. Ten (16%) patients stopped due to leg discomfort only. There was approximately 10% difference in ventilation and exercise time between hyperinflators and nonhyperinflators, and the difference was related to anthropometric characteristics and gender. There were more men among the hyperinflators and more women among the nonhyperinflators.

**Table 1.** Characteristics of the study population.

Variables	Total (*n* = 63)	Women (*n* = 28)	Men (*n* = 35)	*P*‐value
Age (years)	65.7 ± 6.0	64.3 ± 6.2	66.8 ± 5.6	0.089
Pack years	37.2 ± 22.1	30.3 ± 18.7	42.8 ± 23.3	0.028
Height (m)	1.70 ± 0.1	1.63 ± 0.1	1.75 ± 0.1	<0.001
Body mass (kg)	76.0 ± 17.4	68.1 ± 15.7	82.4 ± 16.1	0.001
BMI	26.2 ± 5.0	25.5 ± 5.4	26.8 ± 4.7	0.330
FEV_1_ (L)	1.5 ± 0.6	1.2 ± 0.4	1.6 ± 0.6	0.002
FEV_1_ (% pred)	48.0 ± 14.8	48.9 ± 13.0	47.3 ± 16.2	0.667
FVC (L)	3.1 ± 0.9	2.6 ± 0.6	3.6 ± 0.8	<0.001
FVC (% pred)	82.8 ± 15.3	83.9 ± 16.2	81.9 ± 14.7	0.615
FEV_1_/FVC (%)	46.0 ± 11.1	47.0 ± 10.2	45.2 ± 12.0	0.537
IC (L)	2.2 ± 0.8	1.8 ± 0.5	2.6 ± 0.8	<0.001

Data are presented as mean ± SD. Independent *t*‐test for continuous variables. BMI, body mass index; FEV_1_, forced expiratory volume in 1 sec; FVC, forced vital capacity; IC, inspiratory capacity.

**Table 2.** Peak responses to incremental exercise test on treadmill.

Variables	Total (*n* = 63)	Hyperinflators (*n* = 32)	Nonhyperinflators (*n* = 31)	*P*‐value
Gender, male/female (*n*)	35/28	23/9	12/19	
Exercise time (min)	6.4 ± 2.2	6.6 ± 2.0	6.3 ± 2.4	0.572
VO_2peak_ (L/min)	1.36 ± 0.5	1.48 ± 0.5	1.23 ± 0.5	0.065
VCO_2peak_ (L/min)	1.34 ± 0.67	1.43 ± 0.7	1.25 ± 0.7	0.308
VE_peak_ (L/min)	47.3 ± 19.6	49.3 ± 20.3	45.3 ± 18.9	0.419
HR_peak_ (bpm)	133 ± 19	132 ± 18	134 ± 20	0.711
Dyspnea (Borg Scale)	8.7 ± 1.6	8.8 ± 1.6	8.6 ± 1.6	0.626
Leg discomfort (Borg Scale)	5.5 ± 3.0	5.4 ± 2.5	5.7 ± 3.4	0.666
ΔIC(L)	0.46 ± 0.33	0.72 ± 0.25	0.20 ± 0.15	<0.001
SpO_2_% start	95.9 ± 2.5	95.4 ± 2.7	96.5 ± 2.3	0.083
SpO_2_% end	89.6 ± 5.1	89.2 ± 5.5	90.0 ± 4.7	0.533

Data are presented as mean ± SD, unless otherwise stated. VO_2_, oxygen uptake; VCO_2_, carbon dioxide production; VE, ventilation, tidal volume; HR, heart rate; ΔIC, inspiratory capacity, IC at the start of the test minus IC at the end of the test; SpO_2_, oxygen saturation.

**Figure 3.**
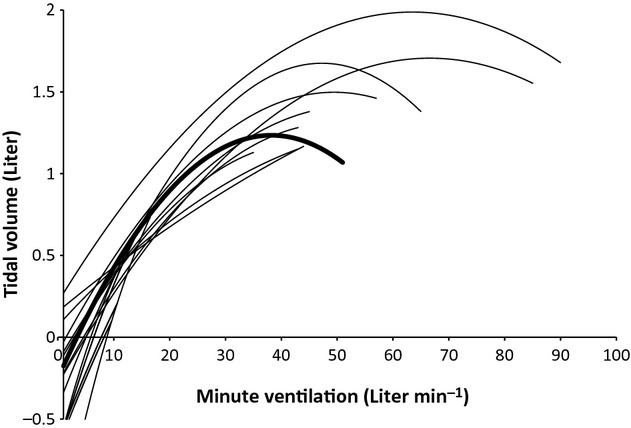
A random set of 14 individual responses (thin lines) and the mean response for the 59 patients (bold line).

In 59 patients, the *P*‐value of the *F*‐statistic for the quadratic model was <0.05 and the *R*^2^ ranged from 0.40 to 0.98 (median of 0.90). Four patients were excluded from further analysis, because in the individual analysis the goodness of fit was not statistically significant. In these patients, the exercise time was short and few data points were available for computing the regression curve. Two of these patients were in GOLD stage III and two in GOLD stage IV. Figure 3 shows a random set of 14 individual responses and the mean response for the 59 patients. The mean of the estimated constant (*a*) was ∢0.18 (SD = 0.44), the mean linear coefficient (*b*) was 0.076 (SD = 0.035), and the mean quadratic coefficient (*c*) was ∢0.00102 (SD = 0.00080).

In the multivariate linear regression analyses, the linear coefficient (*b*) was negatively (*P* = 0.001) and the quadratic coefficient (*c*) positively (*P* < 0.001) related to FEV_1_. Age, gender, height, weight, FVC, IRV, and ΔIC_adj_, were not associated with the curve parameters after adjusting for FEV_1_ (Table 3).

**Table 3.** The relationships between the curve parameters and explanatory variables.

Variable	Unadjusted		Adjusted		
	*B*	*P*‐value	*B*	95% CI	*P*‐value
Curve parameter *a*^1^					
Age	∢0.003	0.761	0.009	∢0.013 to 0.030	0.423
Gender	∢0.088	0.448	∢0.039	∢0.375 to 0.296	0.814
Height	∢0.273	0.666	∢1.209	∢3.497 to 1.079	0.294
Weight	0.001	0.732	9.9 × 10^∢5^	∢0.008 to 0.009	0.981
FEV_1_	0.192	0.059	0.560	0.151 to 0.968	0.008
FVC	0.005	0.938	∢0.184	∢0.477 to 0.110	0.215
ΔIC_adj._	∢0.006	0.187	0.001	∢0.010 to 0.012	0.847
IRV	0.240	0.224	0.066	∢0.448 to 0.580	0.798
Curve parameter *b*^1^					
Age	36.6 × 10^∢5^	0.632	∢0.001	∢0.002 to 0.001	0.439
Gender	0.014	0.133	0.014	∢0.011 to 0.040	0.254
Height	0.015	0.774	0.041	∢0.131 to 0.213	0.633
Weight	∢2.4 × 10^∢5^	0.927	12.3 × 10^∢5^	∢0.001 to 0.001	0.698
FEV_1_	∢0.020	0.016	∢0.053	∢0.083 to ∢0.022	0.001
FVC	∢0.001	0.874	0.016	∢0.006 to 0.038	0.157
ΔIC_adj._	0.001	0.026	20.1 × 10^∢5^	∢0.001 to 0.001	0.622
IRV	∢0.020	0.218	0.007	∢0.032 to 0.045	0.726
Curve parameter *c*^1^					
Age	∢1.4 × 10^∢5^	0.422	1.4 × 10^∢5^	∢1.8^2^/> to 4.7^2^	0.389
Gender	∢9.1 × 10^∢5^	0.668	∢38.1 × 10^∢5^	∢0.001 to 13.5^2^	0.144
Height	0.002	0.193	∢0.001	∢0.004 to 0.003	0.663
Weight	∢0.6 × 10^∢5^	0.334	∢0.2 × 10^∢5^	∢1.5^2^ to 1.1^2^	0.810
FEV_1_	0.001	<0.001	0.001	0.001 to 0.002	<0.001
FVC	27.0 × 10^∢5^	0.026	∢12.6 × 10^∢5^	∢0.001 to 32.5^2^	0.577
ΔIC_adj._	∢1.8 × 10^∢5^	0.026	∢0.6 × 10^∢5^	∢2.3^2^ to 1.1^2^	0.477
IRV	0.001	0.013	∢6.6 × 10^∢5^	∢0.001 to 0.001	0.868

95% confidence interval (CI) examined by linear regression in multivariate analyses (*P *<**0.05). FEV_1_, forced expired volume in 1 sec; FVC, forced vital capacity; ΔIC, inspiratory capacity, IC at the start of the test minus IC at the end of the test; ΔIC_adj._, ΔIC adjusted for resting IC; IRV, inspiratory reserve volume.

* The relationship between *V*_E_ and *V*_T_ was described by a quadratic model (*V*_T_ = *a* + *bV*_E_ + *cV*_E_^2^).

* Values are given multiplied by 10^∢5^.

The *V*_Tmax_ and *V*_E_ at *V*_Tmax_ were calculated from the individual quadratic relationships. In adjusted linear regression analyses, both were related to FEV_1_ (*P* < 0.001), but not to age, gender, height, weight, FVC, and ΔIC.

When using the hyperbolic model, the mean constant was 1.70 (SD = 0.52), and the curvature ∢14.73 (SD = 8.87). The median *R*^2^ was 0.84 (range 0.25–0.95) which was lower than for the quadratic relationship.

## Discussion

The main findings of this study were: (1) The relationship between *V*_T_ and *V*_E_ during incremental exercise could be described by a quadratic model in most COPD patients. (2) The linear and quadratic curve parameters were both related to FEV_1_. With a lower FEV_1_, maximal *V*_T_ was lower and achieved at a lower *V*_E_. (3) Dynamic hyperinflation and IRV were not related to the curve parameters.

When using a curvilinear model to describe the relationship between *V*_E_ and *V*_T_, all observations throughout the incremental exercise test are included in the analysis, and a detailed description of the test from start to end is provided. A limitation with other methods used to describe the relationship between *V*_E_ and *V*_T_ like the Hey et al. (1966) plot, the *V*_T30_ and *V*_Tmax_ (Cotes 1972), and *V*_T_ at given fractions of peak *V*_E_ (Neder et al. 2003), is that all observed data from the exercise test are not included in the analysis. The exercise tests in these studies were done on a cycle ergometer, and in the studies of Cotes (1972) and Hey et al. (1966) the tests were submaximal. Breathing pattern was different with treadmill exercise compared with cycle exercise in a study of young and healthy subjects (Kalsas and Thorsen 2009), but no differences in breathing pattern were observed comparing maximal and submaximal incremental exercise test on a cycle ergometer (Kjelkenes and Thorsen 2010). The *V*_T30_ require that a ventilation of at least 30 L/min is achieved. In our study, 16 of the COPD patients had a peak ventilation below 30 L/min. We did not use a logarithmic model as described by Naranjo et al. (2005), because it does not account for *V*_T_ having a maximal value.

The quadratic model could not be used for all COPD patients in this study. Four patients were excluded from further analysis because the *P*‐value of the *F*‐statistic in the individual analysis was not significant. The exercise time was short and thereby few data points were available for mathematical description of the response in these patients. We considered other mathematical models for all subjects including a hyperbolic model, but with respect to *R*^2^, the parabolic was best. For the four excluded subjects, none of these models were applicable. COPD is a progressive disease and in a general COPD population, not all patients will have the functional capacity to complete an incremental exercise test, which is a strenuous maneuver.

Incomplete expiration leads to accumulation of gas in the lung, and a given ventilatory demand can only be sustained when breathing takes place at a lung volume having a time constant that allows complete respiratory cycles. FEV_1_ is the integrated sum of maximal expiratory flow rates during the first second of a forced exhalation. Maximal expiratory flow rates are determined by airway diameter, compliance of the airway wall, and gas density (Pedersen et al. 1985). COPD is characterized by loss of elastic properties throughout the lung, not specifically located to the airways or the alveolar region (Hogg 2012). In this way, FEV_1_ is related to both resistance and compliance, and thereby to the time constant, which is the product of the two. A relationship between FEV_1_ and the curve parameters determining the breathing pattern is therefore not unexpected.

The TLC is expected to remain unaltered during exercise, and therefore dynamic hyperinflation can be described as a reduction in IC from start to end of the exercise test (∆IC), when end‐expiratory lung volume (EELV) increases (Stubbing et al. 1980; Yan et al. 1997; Vogiatzis et al. 2005). In our study, there was no correlation between FEV_1_ and ∆IC, and as far as we know a relationship between FEV_1_ and ∆IC has not been demonstrated in other studies. We found no relationship between ∆IC and the curve parameters. The hyperinflators in this study were not different from the nonhyperinflators with respect to FEV_1_ in percent of predicted, VO_2peak_, V_Epeak_, and Borg dyspnea score at the end of the test. Desaturation was the same in both groups as well. In young healthy subjects, the individual relationship between *V*_E_ and *V*_T_ has been described satisfactorily by a quadratic relationship (Kalsas and Thorsen 2009), and normal healthy subjects does not hyperinflate during progressive exercise. This may suggest that dynamic hyperinflation is primarily a mechanism for adjusting the time constant of the lung to expiratory flow limitation and is not a determinant of breathing pattern per se.

In healthy subjects, the breathing pattern with respect to *V*_T_ and *B*_f_ has traditionally been considered a load compensating mechanism to minimize the work of breathing (Otis et al. 1950; Widdicombe and Nadel 1963; Poon 1987). However, direct evidence for such a mechanism being operative is lacking. Dynamic hyperinflation and a lower IRV are not load compensating mechanisms and could therefore be independent phenomena. The importance of hyperinflation can, however, not be ignored as it is by itself related to dyspnea, respiratory effort, and work of breathing. The constraint for the expansion of *V*_T_ on the inspiratory side set by TLC, and how close the patients breathe in relationship to TLC, will also be associated with a higher work of breathing.

The participants in this study had participated in a pulmonary rehabilitation program. The patients recruited could therefore be biased to have higher functional capacity than the common COPD population. The distribution among GOLD stages were 32 patients in stage II, 23 in stage III and eight in stage IV, respectively. There were fewer patients with more serious disease as represented by GOLD stage IV and the most severely ill patients were not able to participate in the study. However, 49% of the patients were in GOLD stages III and IV. We therefore assume that our study population is representative for the common COPD patients met in outpatient clinics or in hospitals.

## Conclusion

The curvilinear model provides a method to describe the breathing pattern during exercise in most COPD patients. The curve parameters were related to FEV_1_. With a lower FEV_1_, maximal *V*_T_ was lower and achieved at a lower *V*_E_. Dynamic hyperinflation and IRV were not related to breathing pattern when adjusting for FEV_1_.

## Acknowledgments

The authors thank Eli Nordeide, Lene Svendsen, and Michael Storebø for participation in data collection and for help in organizing the study. We also wish to acknowledge the patients who participated in the study.

## Conflict of Interest

None declared.
